# Triple isotope variations of monthly tap water in China

**DOI:** 10.1038/s41597-020-00685-x

**Published:** 2020-10-12

**Authors:** Chao Tian, Lixin Wang, Wenzhe Jiao, Fadong Li, Fuqiang Tian, Sihan Zhao

**Affiliations:** 1 Key Laboratory of Ecosystem Network Observation and Modeling, Institute of Geographic Sciences and Natural Resources Research, Chinese Academy of Sciences, Beijing, 100101 China; 2 Department of Earth Sciences, Indiana University-Purdue University Indianapolis (IUPUI), Indianapolis, IN 46202 USA; 3 University of Chinese Academy of Sciences, Beijing, 100049 China; 4 Department of Hydraulic Engineering, State Key Laboratory of Hydroscience and Engineering, Tsinghua University, Beijing, 100084 P.R. China

**Keywords:** Hydrology, Environmental sciences

## Abstract

Tap water isotopic compositions could potentially record information on local climate and water management practices. A new water isotope tracer ^17^O-excess became available in recent years providing additional information of the various hydrological processes. Detailed data records of tap water ^17^O-excess have not been reported. In this report, monthly tap water samples (n = 652) were collected from December 2014 to November 2015 from 92 collection sites across China. The isotopic composition (δ^2^H, δ^18^O, and δ^17^O) of tap water was analyzed by a Triple Water Vapor Isotope Analyzer (T-WVIA) based on Off-Axis Integrated Cavity Output Spectroscopy (OA-ICOS) technique and two second-order isotopic variables (d-excess and ^17^O-excess) were calculated. The geographic location information of the 92 collection sites including latitude, longitude, and elevation were also provided in this dataset. This report presents national-scale tap water isotope dataset at monthly time scale. Researchers and water resource managers who focus on the tap water issues could use them to probe the water source and water management strategies at large spatial scales.

## Abstract (summary)

**Table:** 

Measurement(s)	Isotope • oxygen-17 atom • oxygen-18 atom • stable hydrogen isotopes • tap water isotopic composition
Technology Type(s)	laser absorption spectroscopy • stable isotope analysis
Factor Type(s)	tap water collection site • month of tap water sample collection
Sample Characteristic - Environment	tap water
Sample Characteristic - Location	China

Machine-accessible metadata file describing the reported data: 10.6084/m9.figshare.12923681

## Background & Summary

Stable isotopes of hydrogen and oxygen have been widely used to identify plant water uptake depths, partition evapotranspiration, and separate hydrographs^1–7^. Such applications rely on different isotopic compositions of different water pools and the isotope difference is fundamentally caused by isotope fractionation. There are two major isotope fractionation processes: equilibrium fractionation and kinetic fractionation when water vapor, liquid, or ice crystals are converted into each other. Equilibrium fractionation is mainly affected by different saturation vapor pressure (e.g., liquid condensation)^8,9^ and kinetic fractionation is mainly affected by diffusivities (e.g., evaporation and solid condensation at supersaturation)^9,10^.

^17^O is the least abundant (0.038%) oxygen isotope and can be used as a new tracer in meteorological and hydrological studies. Due to the advances of high-precision analytical methods^11–13^, ^17^O-excess (^17^O-excess = ln (δ^17^O + 1) − 0.528 x ln (δ^18^O + 1)), another important second-order isotope like d-excess (d-excess = δ^2^H – 8 x δ^18^O), becomes available to probe hydrological processes^11,12,14^. Taking precipitation formation as an example, the δ^2^H, δ^18^O, δ^17^O, and d-excess are all sensitive to both temperature and relative humidity^10,15,16^. However, ^17^O-excess is theoretically not affected by temperature and only affected by relative humidity between 10 °C to 45 °C because of the similar temperature sensitivity between δ^18^O and δ^17^O^17,18^. Therefore, combing ^17^O-excess and ^18^O measurements could separate the temperature (not affecting ^17^O-excess) and relative humidity (affecting both ^17^O-excess and ^18^O) effect on oxygen isotopes. ^17^O-excess can also be used to identify spectral contamination and improve direct vapor equilibration in plant and soil analysis^19^. According to the relationship between δ′^18^O and δ′^17^O (i.e., the slope of 1000 x ln (δ^18^O + 1) and 1000 x ln (δ^17^O + 1)), synoptic drought related to EI Nino and local drought is distinguishable^20^. Fog and dew are also differentiated using the δ′^18^O and δ′^17^O relationship at the Namib Desert^21^. Moreover, based on the conceptual evaporation model, the relationship between δ′^18^O and δ′^17^O, and the relationships between ^17^O-excess and δ′^18^O (or d-excess)) are used to estimate whether water (e.g., precipitation, river waters, and lake waters) is affected by equilibrium fractionation or kinetic fractionation associated with evaporation^14,17,22–28^. Up to now, the studies of water ^17^O-excess variations at large spatiotemporal distribution have mainly focused on snow and ice cores in high-latitude regions^29–36^, where ^17^O-excess of snow is sensitive to temperature because of kinetic fractionation associated with supersaturation conditions under extremely cold condition (−80 to −15 °C)^29,31,32^. There are only few studies focused on the mid-latitude regions^24,25,37,38^.

The Intergovernmental Panel on Climate Change reported extending durations of severe droughts, increasing surface temperatures, and decreasing rainfall^39,40^. Thus, tap water, as an essential part of the domestic water use, should be paid more attention due to the trend of water scarcity and severe water pollution. The isotope variations of tap water could reveal the regional water supply sources, and reflect water-resource management strategies that integrate human geography, climate and socio-economic development^1,41^. The tap water in some regions can be used as a precipitation proxy to study the local precipitation^41,42^, while other regions may be supplied from inter-basin water transfers, deep groundwater or montane snowmelt^1,43^. The water resources in the north of China are less than those in the south due to special geographical location, climate change, extensive water-intensive economic activities, and population growth^44–46^. Therefore, the spatiotemporal distribution of tap water isotopes in China are needed to better understand water sources, thus informing water resource management.

To our best knowledge, there is no monthly tap water isotope dataset including ^17^O-excess publicly available. Here, we provide monthly isotope dataset (δ^2^H, δ^18^O, δ^17^O, d-excess, and ^17^O-excess) of tap water in China collected between December 2014 to November 2015. The instrument operation (δ^2^H, δ^18^O, and δ^17^O) using Triple Water Vapor Isotope Analyzer (T-WVIA-45-EP; Los Gatos Research Inc. (LGR), Mountain View, CA, USA) based on Off-Axis Integrated Cavity Output Spectroscopy (OA-ICOS) technique has been described in details in our previous studies^24,37^, as well as the detailed description of ^17^O-excess quality control method. We have published the tap water isotopic variations in Tian *et al*.^47^. In this new dataset, we present the first publicly available monthly tap water isotope dataset to fill the gap in global tap water isotope datasets, especially for ^17^O-excess, which would be used to study water resource issues in the sustainable development of human societies.

## Methods

### Sample collections

The monthly tap water samples across China were collected in 2015 (from December 2014 to November 2015) by Zhao *et al*.^48^, and conventional isotopes (δ^2^H and δ^18^O) were measured in Hydrology Laboratory of Tsinghua University. To obtain ^17^O-excess values, the samples were delivered to the IUPUI (Indiana University-Purdue University Indianapolis) Ecohydrology Lab to measure δ^18^O and δ^17^O (δ^2^H was also measured simultaneously). 652 samples from 92 sites in China were measured (Online-only Table 1), which have been reported by Tian *et al*.^47^. In here, we reported the detailed geographical location and monthly isotopic variations especially for ^17^O-excess values.

### Isotope measurements and ^17^O-excess data processing

The details of the measurement process have been described by Tian *et al*.^37,47^. In brief, each sample was run at 1 Hz for 2 min under 13000 ppm to attain 120 data points using a Triple Water Vapor Isotope Analyzer (T-WVIA-45-EP, Los Gatos Research Inc. (LGR), Mountain View, CA, USA; preheated to 50 °C) coupled to a Water Vapor Isotope Standard Source (WVISS, LGR, Mountain View, CA, USA; preheated to 80 °C)^49^. To avoid memory effects between samples, the WVISS nebulizer was first purged for at least two minutes, and then the “stabilize” option of the device was turned on for two minutes to expel residual air inside the vaporizing chamber. The operation is different from the liquid water analyzer as described in other studies^50,51^. LGR#1 to LGR#5, as working standards with known and wide range of isotopic composition, were analyzed after every five tap water samples to ensure the accuracy of the T-WVIA performance. Furthermore, normalizing all of the isotope ratios using Vienna Standard Mean Ocean Water (VSMOW) and Standard Light Antarctic Precipitation (SLAP) to reduce differences between laboratories once a day^12,52^.

Accurate ^17^O-excess value of each sample (120 data points) require two steps for quality control. Firstly, calculated λ value (λ = ln (δ^17^O + 1)/ln (δ^18^O + 1) of each data point, the same as theoretical kinetic and equilibrium fractionation coefficient (θ) between liquid and vapor, should be between 0.506 and 0.530^2,53^. Secondly, the calculated ^17^O-excess value of each data point should be between −100 per meg and +100 per meg (1 per meg = 0.001‰), which is the range for almost all of the ^17^O-excess values of global precipitation^2,17,23,25,54^. The data points that meet the above two conditions were averaged to obtain the ^17^O-excess value for that sample.

## Data Records

**Fig. 1:**
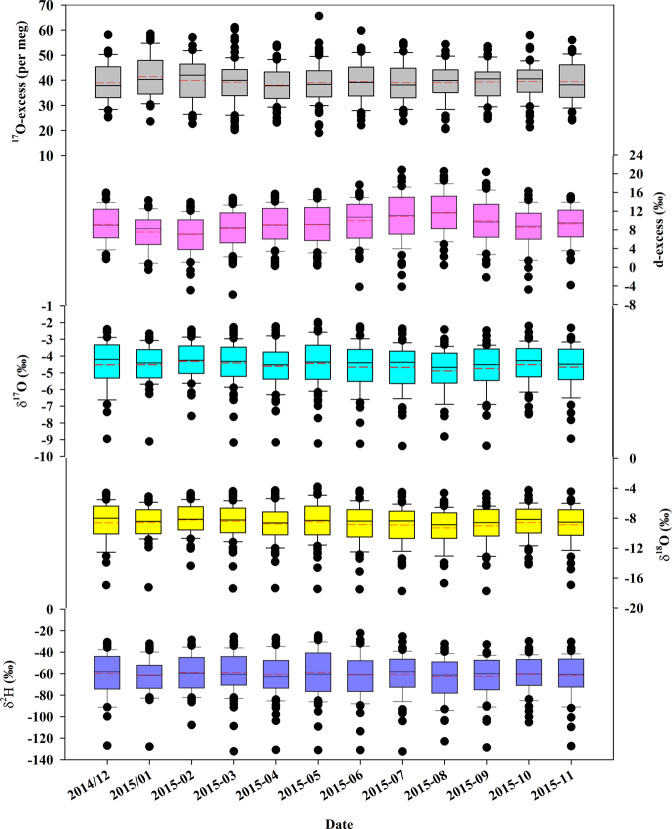
δ^2^H, δ^18^O, and δ^17^O, as well as the d-excess and ^17^O-excess values of monthly tap water from December 2014 to November 2015 in 92 collection sites across China.

**Fig. 2:**
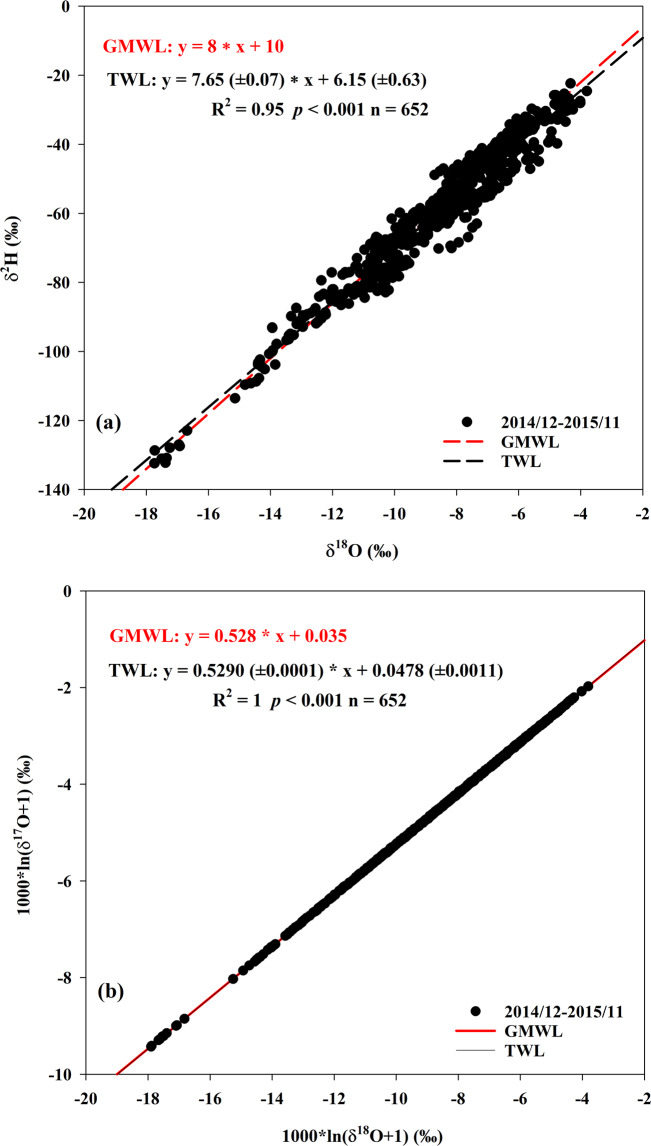
The relationships between monthly tap water δ^18^O and δ^2^H (**a**) as well as δ′^18^O and δ′^17^O (**b**) for all the samples.

Monthly tap water isotope database is archived in PANGAEA in a single table including 652 rows and 10 columns^55^. Each row presents a monthly tap water event at one site. Each column corresponds to the geographic location information (including latitude, longitude, and elevation) and isotope variables including three measured individual stable isotopes (δ^2^H, δ^18^O, and δ^17^O) and two calculated second-order isotopic variables (d-excess and ^17^O-excess). A summary of the tap water in 2015 for 92 sites in China is presented in Table 1. The database spanned over 30.21° in latitude (from 20.03°N to 50.24°N) and 51.50° in longitude (from 75.99°E to 127.49°E). The elevation varied from 5 m to 3657 m with a mean value of 708 m. Fig. 1 depicts the distribution of monthly stable isotopes. The δ^2^H values varied from −132.40‰ to −22.36‰ with a mean value of −60.52 ± 19.54‰ (Table 1). The δ^18^O values varied from −17.74‰ to −3.8‰ with a mean value of −8.72 ± 2.49‰. The δ^17^O values varied from −9.38‰ to −1.97‰ with a mean value of −4.58 ± 1.32‰. The d-excess values varied from −5.9‰ to 20.8‰ with a mean value of 9.2 ± 4.5‰. The ^17^O-excess values varied from 19 to 66 per meg with a mean value of 39 ± 8 per meg. The tap water line (TWL) in China between δ^18^O and δ^2^H based on the 652 tap water samples within one year was δ^2^H = 7.65 (±0.07) x δ^18^O + 6.15 (±0.63) (R^2^ = 0.95, *p* < 0.001), which is close to the Global Meteoric Water Line (GMWL, δ^2^H = 8 x δ^18^O + 10) (Fig. 2a). The tap water line (TWL) between δ′^18^O and δ′^17^O was ln (δ^17^O + 1) = 0.5290 (±0.0001) x ln (δ^18^O + 1) + 0.000048 (±0.000001) (R^2^ = 1, *p* < 0.001), similar to the GMWL for oxygen (ln (δ^17^O + 1) = 0.528 x ln (δ^18^O + 1) + 0.000035, normalized to the VSMOW-SLAP scale^25,54^ (Fig. 2b).

**Table 1:** Summary of the monthly tap water record over one year (from December 2014 to November 2015) of 92 collection sites in China.

	Latitude (^o^)	Longitude (^o^)	Elevation (m a.s.l)	δ^2^H (‰)	δ^18^O (‰)	δ^17^O (‰)	d-excess (‰)	^17^O-excess (per meg)
Mean	33.24	109.76	708	−60.52	−8.72	−4.58	9.2	39
Standard deviation	6.71	10.63	917	19.54	2.49	1.32	4.5	8
Maximum	50.24	127.49	3657	−22.36	−3.8	−1.97	20.8	66
Minimum	20.03	75.99	5	−132.4	−17.74	−9.38	−5.9	19
Range	30.21	51.5	3652	110.04	13.94	7.41	26.7	47

## Technical Validation

The precision of our measurement (δ^2^H, δ^18^O, δ^17^O, and ^17^O-excess) have been described in our previous studies using two international standards (SLAP and Greenland Ice Sheet Precipitation) and the five working standards from LGR, as well as comparing the reported precision in others literature^37,47^. They demonstrated that the precision of our OA-ICOS technique is comparable with other methods including IRMS technique^25,31,32,34,52,54^, CRDS method^12,38^, and other type of OA-ICOS water analyzer^11^.

## Online-only Table

**Online-only Table 1:** Summary of geographic location information and δ^2^H, δ^18^O, δ^17^O, d-excess, and ^17^O-excess annual values for 92 tap water collection sites in China in 2015 (collected between December 2014 to November 2015).

Site	Latitude (^o^)	Longitude (^o^)	Elevation (m a.s.l)	δ^2^H (‰)	δ^18^O (‰)	δ^17^O (‰)	d-excess (‰)	^17^O-excess (per meg)
Hehei	50.24	127.49	139	−106.93	−14.45	−7.63	8.7	26
Harbin	45.74	126.64	147	−92.10	−12.71	−6.70	9.6	28
Karamay	45.60	84.86	410	−77.07	−11.64	−6.12	16.0	40
Urumchi	43.79	87.61	883	−72.57	−10.70	−5.62	13.0	43
Aksu	41.17	80.26	1107	−64.14	−9.42	−4.94	11.2	41
Korla	41.76	86.13	940	−57.22	−8.08	−4.24	7.5	38
Kashgar	39.47	75.99	1296	−91.03	−13.18	−6.94	14.4	38
Jiuquan	39.74	98.51	1470	−74.79	−10.80	−5.67	11.6	45
Delingha	37.37	97.36	2995	−61.71	−9.67	−5.08	15.7	41
Golmud	36.43	94.89	2803	−66.53	−10.03	−5.27	13.7	43
Xining	36.61	101.79	2261	−47.10	−7.99	−4.19	16.8	39
Lanzhou	36.07	103.75	1543	−71.95	−10.24	−5.38	10.0	42
Baiyin	36.54	104.18	1713	−50.21	−7.97	−4.17	13.5	41
Baotou	40.67	109.85	1068	−70.28	−9.65	−5.06	6.9	47
Hohhot	40.82	111.66	1057	−77.61	−10.81	−5.69	8.9	37
Linhe	40.76	107.39	1040	−75.50	−9.85	−5.18	3.3	33
Yinchuan	38.47	106.27	1113	−84.92	−11.98	−6.30	10.9	40
Yulin	38.30	109.76	1121	−60.53	−7.62	−3.99	0.4	41
Taiyuan	37.87	112.57	797	−64.00	−8.59	−4.50	4.7	43
Jinzhong	37.68	112.75	800	−60.35	−8.29	−4.35	5.9	38
Shijiazhuang	38.05	114.49	80	−51.99	−6.74	−3.52	2.0	48
Anyang	36.10	114.35	78	−60.99	−8.42	−4.42	6.4	38
Pingliang	35.54	106.68	1365	−69.01	−10.17	−5.35	12.4	38
Ulanhot	46.07	122.07	273	−81.57	−10.91	−5.73	5.7	39
Xilinhot	43.94	116.07	988	−82.45	−10.35	−5.45	0.3	32
Tongliao	43.61	122.26	181	−78.72	−10.14	−5.33	2.4	40
Changchun	43.89	125.32	227	−79.96	−10.36	−5.43	2.9	48
Chifeng	42.27	118.95	572	−74.62	−9.70	−5.10	3.0	33
Shenyang	41.80	123.41	50	−67.36	−9.22	−4.85	6.4	33
Chengde	40.97	117.92	361	−62.37	−8.26	−4.33	3.7	37
Dandong	40.14	124.38	18	−58.76	−8.48	−4.46	9.1	33
Tianjin	39.13	117.20	9	−55.09	−6.98	−3.66	0.7	33
Tangshan	39.63	118.20	23	−57.46	−7.86	−4.12	5.4	34
Baoding	38.86	115.50	21	−63.65	−8.69	−4.56	5.9	43
Cangzhou	38.31	116.86	8	−77.63	−10.48	−5.51	6.2	39
Dalian	38.92	121.60	21	−52.32	−6.96	−3.65	3.4	37
Hengshui	37.73	115.71	26	−79.78	−10.90	−5.73	7.4	35
Dongying	37.46	118.50	5	−51.74	−6.60	−3.45	1.1	46
Yantai	37.54	121.38	43	−46.76	−6.19	−3.24	2.8	38
Weifang	36.70	119.11	28	−58.69	−7.98	−4.19	5.2	33
Lhasa	29.66	91.13	3657	−129.16	−17.30	−9.14	9.2	31
Gannan	35.20	102.51	3012	−68.08	−10.15	−5.33	13.1	40
Dingxi	35.58	104.62	1905	−69.81	−10.37	−5.45	13.2	37
Longnan	33.39	104.93	1174	−69.02	−10.43	−5.49	14.4	33
Chengdu	30.66	104.08	497	−83.92	−12.27	−6.46	14.2	38
Nyingchi	29.58	94.48	3310	−98.39	−13.81	−7.28	12.1	33
Xichang	27.90	102.27	1563	−72.35	−9.06	−4.75	0.1	40
Panzhihua	26.55	101.70	1064	−104.56	−14.16	−7.47	8.7	30
Baoshan	25.12	99.17	1667	−68.50	−9.71	−5.10	9.2	40
Kunming	25.04	102.70	1907	−82.69	−11.24	−5.91	7.2	35
Qujing	25.50	103.79	1868	−71.13	−9.40	−4.93	4.1	41
Simao	22.80	100.98	1336	−62.51	−8.70	−4.57	7.1	36
Wenshan	23.37	104.24	1268	−64.86	−9.07	−4.77	7.7	33
Tianshui	34.58	105.72	1176	−60.05	−9.08	−4.77	12.6	36
Zhengzhou	34.76	113.65	106	−58.94	−8.29	−4.35	7.3	35
Kaifeng	34.79	114.35	70	−56.04	−7.69	−4.03	5.5	38
Hanzhong	33.08	107.03	515	−54.52	−8.43	−4.42	12.9	42
Xianyang	34.34	108.71	384	−83.41	−11.45	−6.02	8.2	39
Enshi	30.27	109.48	421	−46.74	−7.37	−3.85	12.3	46
Wuhan	30.57	114.29	16	−57.36	−8.62	−4.52	11.6	41
Chongqing	29.56	106.51	157	−42.50	−6.73	−3.52	11.3	43
Yueyang	29.37	113.10	46	−28.65	−4.63	−2.40	8.4	47
Changsha	28.20	112.98	54	−30.02	−5.18	−2.69	11.4	45
Bijie	27.31	105.28	1478	−59.13	−8.87	−4.65	11.8	39
Tongren	27.72	109.19	274	−43.54	−7.24	−3.79	14.4	42
Huaihua	27.55	109.95	227	−40.66	−6.65	−3.48	12.5	34
Xiangtan	27.87	112.92	43	−30.91	−5.12	−2.67	10.1	37
Guiyang	26.58	106.71	1073	−49.64	−7.43	−3.89	9.8	38
Guilin	25.28	110.29	160	−37.19	−6.32	−3.30	13.4	41
Zaozhuang	34.87	117.56	80	−45.88	−6.11	−3.19	3.0	41
Xuzhou	34.27	117.19	35	−76.39	−10.50	−5.52	7.6	40
Suzhou	33.64	116.97	37	−57.40	−8.05	−4.22	7.0	44
Yancheng	33.39	120.14	5	−37.03	−5.20	−2.70	4.6	44
Nantong	32.02	120.86	11	−46.42	−7.09	−3.71	10.3	36
Hefei	31.86	117.28	22	−37.53	−5.57	−2.90	7.0	48
Ma’anshan	31.72	118.48	29	−48.24	−7.22	−3.77	9.5	44
Shanghai	31.24	121.47	16	−30.88	−4.74	−2.46	7.0	44
Shaoxing	30.01	120.57	11	−48.50	−7.54	−3.95	11.8	36
Hangzhou	30.27	120.16	18	−40.01	−6.43	−3.34	11.4	55
Quzhou	28.96	118.87	79	−41.95	−6.76	−3.54	12.2	40
Lishui	28.45	119.92	64	−46.42	−7.37	−3.86	12.6	42
Fuzhou	26.08	119.30	18	−40.69	−6.56	−3.43	11.8	41
Longyan	25.11	117.03	365	−39.06	−6.59	−3.44	13.6	46
Liuzhou	24.31	109.40	65	−35.26	−6.17	−3.23	14.1	36
Shaoguan	24.81	113.61	65	−39.24	−6.60	−3.45	13.6	45
Xiamen	24.46	118.09	31	−40.84	−6.50	−3.40	11.2	39
Bose	23.90	106.61	141	−60.25	−8.91	−4.67	11.0	41
Guangzhou	23.12	113.26	28	−37.28	−5.99	−3.12	10.7	47
Nanning	22.81	108.31	80	−55.67	−8.06	−4.22	8.8	42
Shenzhen	22.56	114.11	8	−35.48	−5.69	−2.97	10.0	37
Zhanjiang	21.19	110.40	17	−37.95	−5.58	−2.90	6.7	46
Haikou	20.03	110.35	15	−44.55	−6.60	−3.45	8.2	44

The detailed geographical information of each sampling locations can be found in Zhao et al.^1^.
